# Gender and intention to leave healthcare during the COVID-19 pandemic among U.S. healthcare workers: A cross sectional analysis of the HERO registry

**DOI:** 10.1371/journal.pone.0287428

**Published:** 2023-06-16

**Authors:** Rachel Apple, Emily C. O’Brien, Nancy M. Daraiseh, Haolin Xu, Russell L. Rothman, Mark Linzer, Laine Thomas, Christianne Roumie

**Affiliations:** 1 Departments of Medicine and Pediatrics, Vanderbilt University Medical Center, Nashville, Tennessee, United States of America; 2 Duke Clinical Research Institute and Department of Population Health Sciences, Duke University School of Medicine, Durham, North Carolina, United States of America; 3 Department of Pediatrics, University of Cincinnati College of Medicine, Cincinnati Children’s Hospital Medical Center, Cincinnati, Ohio, United States of America; 4 Department of Health Policy, Vanderbilt University Medical Center, Nashville, Tennessee, United States of America; 5 Department of Medicine, Hennepin Healthcare and University of Minnesota, Minneapolis, Minnesota, United States of America; 6 Duke Clinical Research Institute and Department of Biostatistics & Bioinformatics, Duke University School of Medicine, Durham, North Carolina, United States of America; 7 Veteran Administration Tennessee Valley VA Health Care System Geriatric Research Education Clinical Center (GRECC), Nashville, Tennessee, United States of America; Stamford Health, UNITED STATES

## Abstract

**Importance:**

The COVID-19 pandemic stressed the healthcare field, resulting in a worker exodus at the onset and throughout the pandemic and straining healthcare systems. Female healthcare workers face unique challenges that may impact job satisfaction and retention. It is important to understand factors related to healthcare workers’ intent to leave their current field.

**Objective:**

To test the hypothesis that female healthcare workers were more likely than male counterparts to report intention to leave.

**Design:**

Observational study of healthcare workers enrolled in the Healthcare Worker Exposure Response and Outcomes (HERO) registry. After baseline enrollment, two HERO ‘hot topic’ survey waves, in May 2021 and December 2021, ascertained intent to leave. Unique participants were included if they responded to at least one of these survey waves.

**Setting:**

HERO registry, a large national registry that captures healthcare worker and community member experiences during the COVID-19 pandemic.

**Participants:**

Registry participants self-enrolled online and represent a convenience sample predominantly composed of adult healthcare workers.

**Exposure(s):**

Self-reported gender (male, female).

**Main outcome:**

Primary outcome was intention to leave (ITL), defined as having already left, actively making plans, or considering leaving healthcare or changing current healthcare field but with no active plans. Multivariable logistic regression models were performed to examine the odds of intention to leave with adjustment for key covariates.

**Results:**

Among 4165 responses to either May or December surveys, female gender was associated with increased odds of ITL (42.2% males versus 51.4% females reported intent to leave; aOR 1.36 [1.13, 1.63]). Nurses had 74% higher odds of ITL compared to most other health professionals. Among those who expressed ITL, three quarters reported job-related burnout as a contributor, and one third reported experience of moral injury.

**Conclusions and relevance:**

Female healthcare workers had higher odds of intent to leave their healthcare field than males. Additional research is needed to examine the role of family-related stressors.

**Trial registration:**

ClinicalTrials.gov identifier NCT04342806.

## Background

The COVID-19 pandemic impacted healthcare workers in many ways, causing strain on the healthcare system. At the start of the pandemic, many healthcare workers cared for critically ill patients, creating long work hours and work stress for themselves and their immediate family due to increased exposure and risk of contracting the SARS CoV2 virus which causes COVID- 19 [1, 2]. The COVID-19 pandemic also created a shift in personal and home responsibilities, particularly among those with children and elderly persons in the home whose typical care or activities were disrupted. Thus, essential healthcare workers were placed in situations which necessitated high-stress vocational work and unique stresses in dependent care [3–7]. As a result, sections of the healthcare workforce experienced a worker exodus at the onset and throughout the pandemic [8, 9]. Furthermore, healthcare workers continue to leave, leading to major projected shortages in the healthcare field in the months and years to come [10].

Intention to leave one’s job refers to a person’s thoughts related to job separation and is considered a proxy predictor of voluntary turnover among workers [11]. Causes for turnover among healthcare workers include control of one’s schedule, job satisfaction [12], work environment [13], and burnout or emotional exhaustion [14]. Few studies have examined gender differences related to work-life balance and home-duties among healthcare workers [15, 16]. In a 2018 study, Ly et al. reported that married female physicians with children spent significantly more time daily on household activities and childcare duties than their male counterparts [14]. Following the onset of the COVID-19 pandemic, several studies highlighted gender differences in job productivity and promotion in medical academia, most of which were attributed to the unequal distribution of additional home and childcare duties in the midst of school and daycare closures [4, 6, 17, 18].While several studies examined the relationship between the COVID-19 pandemic and healthcare worker attrition, few have examined the impact of gender on intention to leave and among multiple roles within healthcare [4, 17, 19–23].

Smaller studies report that some groups seemed to be leaving healthcare at disproportionate rates, including females and nurses. We sought to understand these patterns and address the knowledge gap related to the longitudinal effects of the pandemic on the workforce. The use of a large, national registry such as the Healthcare Worker Exposure Response and Outcomes (HERO) registry permits further investigation of these relationships in the setting of the COVID pandemic.

Our aim was to evaluate whether female healthcare workers would be more likely than male healthcare workers to report intention to leave or change healthcare fields in the HERO registry, a large national registry that captures healthcare worker experiences during the COVID-19 pandemic. We further sought to understand the experience of burnout and moral injury in those who expressed intent to leave.

## Methods

### Data sources, study design and population

The HERO Registry (ClinicalTrials.gov Identifier NCT04342806) was approved by the WIRB-Copernicus Group Institutional Review Board (WCG IRB). All participants provided written informed consent to participate in the registry. This manuscript was prepared according to the STROBE guidelines for observational studies. Details on governance, recruitment and data collection have been described previously [24].

In brief, participant recruitment was conducted nationally, in collaboration with PCORnet®, the National Patient-Centered Clinical Research Network, and involved institution-wide emails from participating sites, print advertisements, social media advertisements, newsletters, and professional society solicitations. Participants completed informed consent and self-enrolled in the study online. The Registry population represents a convenience sample predominantly composed of adult healthcare workers, although community members were also eligible to enroll in the HERO registry. After the baseline data collection of COVID-19 exposure and experiences in May 2020, there were two additional HERO hot topic survey waves which were included in this analysis which occurred in May 2021 and December, 2021. ‘Hot topic’ surveys were administered for several months to ascertain the opinion of registry participants regarding timely topics related to COVID-19 (e.g., moral injury, vaccine willingness). Unique participants were included if they responded to the baseline surveys and at least one of the hot topic survey waves. All data collected in the study are participant-reported data [25]. Data for this study was collected up through December 2021, analyzed in the summer of 2022, and submitted for publication in fall 2022.

### Primary exposure and outcome variables

The primary exposure of interest was self-reported gender captured during the baseline assessment (male, female). There were 23 participants who identified as transgender/gender expansive (14), not listed (3), or missing/prefer not to answer (6), and these participants were excluded from the final analyses. In the HERO hot topic waves, participants were asked “As a result of the COVID-19 pandemic, have you thought about leaving healthcare?” Response outcomes included: (0) No I have not thought about leaving; (1) I have thought about but not actively planning to leave healthcare; (2) I have thought about but not actively planning to change my field or area of healthcare; (3) I have already left the healthcare field; (4) I have already changed my field or area of healthcare; (5) I am actively making plans to leave the healthcare field; (6) I am actively making plans to change my field or area of healthcare.

The outcome variable was categorized as a binary outcome where “intention to leave/left” = yes for all participants with a response of 1–6 (those who had left, thinking about leaving or were actively planning to leave healthcare or their field or area of healthcare versus no intention to leave). In a sensitivity analysis, we created an alternative outcome dichotomizing “intention to leave” as those who had left or were actively planning to leave healthcare or their field or area of healthcare versus those with no intention to leave or thinking about leaving but not actively planning (options 3–6 above).

In both the May and December 2021 HERO hot topic surveys, participants who reported any intention to leave were asked about possible contributors, including burnout and moral injury. The item asked, “What factors have contributed to your thoughts about leaving or changing your field/area in healthcare (select all that apply)?” Response options included: Job-related burnout; Job-related moral injury; Concerns about infection; Financial stressors; Family reasons; Availability of other professional opportunities. Burnout was defined as “a state of physical or emotional exhaustion as a result of chronic workplace stress.” Moral injury was defined as “a state of psychological distress as a result of acting or witnessing behaviors that go against your values and/or moral beliefs.”

### Covariates

Baseline covariates included age group of respondent in years (18–29, 30–49, 50–64, 65+); race/ ethnicity (White, Black/African-American, Hispanic/Latino, Asian/Pacific Islander, Other/mixed/prefer not to answer); region of the country (Northeast, Midwest, South, West); Healthcare worker role (Independent providers (Physician, Physician assistant, Nurse practitioner); Nurse (Registered Nurse or Licensed Practical Nurse); Emergency Worker (Paramedic/EMS); Ancillary clinical workers (Other Practitioners, Tech/Support and Other healthcare workers); Non clinical (Administrators or Research worker) and survey wave (May or December). Other covariates included family composition (adults, children, senior; 3 levels: yes/no/missing), healthcare facility type, healthcare location within facility (high infection risk unit, other unit, technical clinical service, non-clinical service, other) and weekly work hours (“before pandemic” and “in the past week”).

### Statistical analysis

Descriptive statistics included the medians, ranges and proportions for each gender. Baseline characteristics and study outcomes used Chi-square test of proportions for univariate comparisons. We tested the proportional odds assumption to allow for the outcome to be analyzed ordinally. The outcome was non proportional between levels and the assumptions were not met. Thus, the primary analysis dichotomized the responses into a binary outcome as delineated above. Multivariable logistic regression modeled the primary outcome, intention to leave or change fields, by gender adjusting for the above covariates of age, race/ethnicity, healthcare worker role, survey wave, family composition, self-reported caretaker status, healthcare facility, healthcare location, and weekly work hours. We tested for effect measure modification between gender and healthcare worker role; test for an interaction was not statistically significant for the primary (p = 0.489) or the sensitivity ITL endpoints (p = 0.152) and these were not included in the models. Participants who answered the survey in both May and Dec contributed repeated measures, with the primary outcome measured separately at each time point. Multiple responses within the same participant were addressed in the models using GEE (with exchangeable working correlation matrix).

We did multiple exploratory analyses. Among those who reported intention to leave, we evaluated the proportions of burnout and moral injury by gender and across gradations of intent to leave (left or active planning versus thoughts but no active planning). Additionally, we determined if caregiver status (Caregiver defined as responding “Yes” to question: “Do you routinely look after a child or a sick, elderly, or disable person as caregiver?”) was associated with intention to leave. Adjusted Odds Ratios (aOR) and 95% Confidence Intervals (95% CI) are reported.

## Results

### Sample selection and characteristics

**Fig 1:**
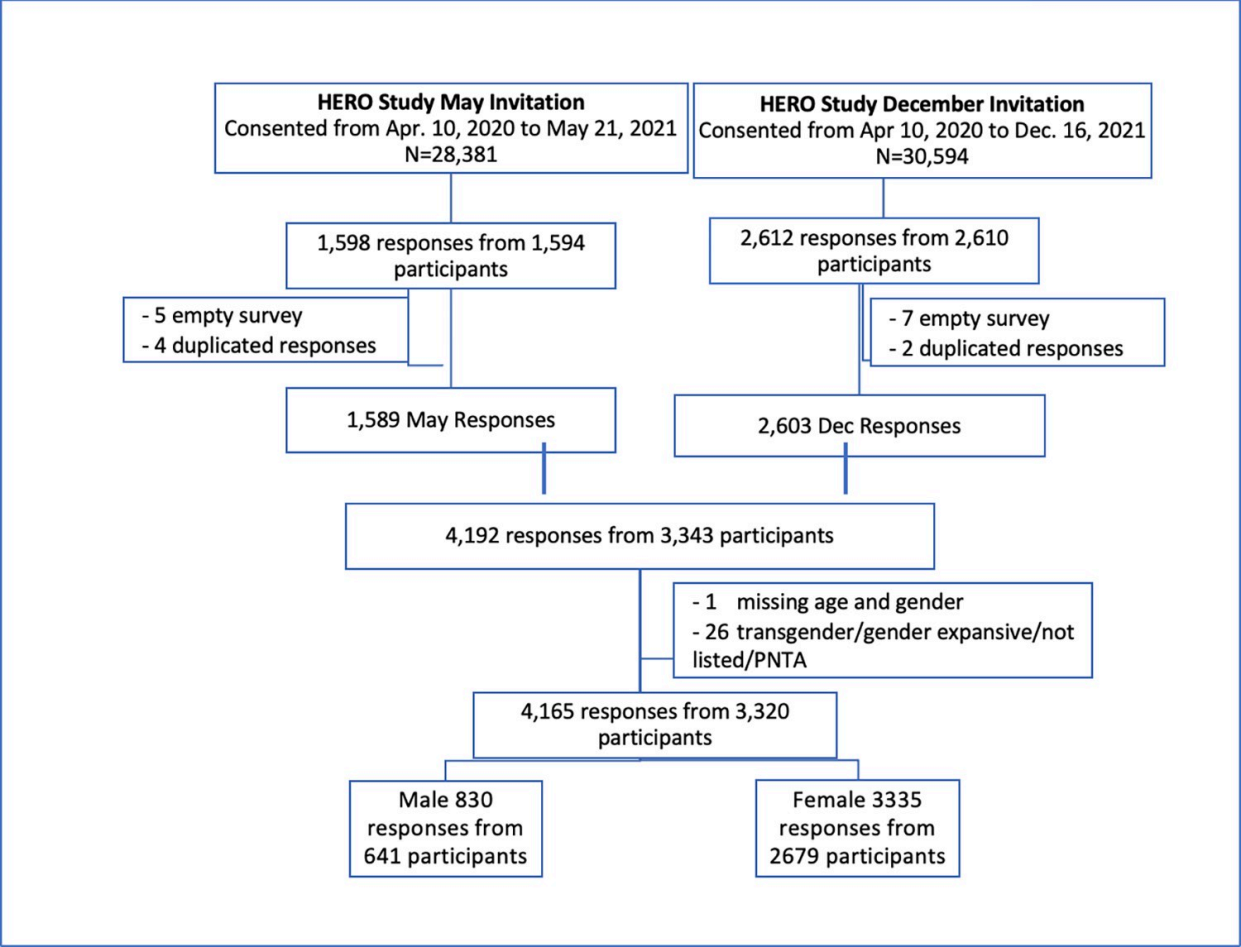
Flow chart of participants.

Overall, there were 3320 respondents to both the baseline and one of the two hot topic waves (May or December 2021), of which 641 (19.3%) were male and 2679 (80.7%) were female (**Fig 1**). The May 2021 survey included 2584 respondents (503 male, 2081 female) and the December 2021 survey included 1581 respondents (327 male, 1254 female). There were 845 participants who completed both the May and the December 2021 surveys, of which 189 were male and 656 were female. The median age of all respondents from either survey was 42.0 years (Interquartile range [IQR] 34, 52 years). The majority of respondents self-identified as white (81.9%). The distribution of respondents’ residence by United States geographic region was 23.3% Northeast, 23.5% Midwest, 41.2% South, and 12.0% West. Among respondents’ healthcare role 35.7% vs 17.4% of males and females identified as independent clinical providers (physician or advanced practice provider). There were 11.5% versus 32.6% males and females identified as nurses. Prior to the pandemic 72.5% of male vs 56.4% of female respondents reported working 40+ hours per week. Almost 10% of females reported being a caretaker of a dependent including children or elderly adults (**Table 1**).

**Table 1:** Respondent characteristics.

	Overall	Male	Female
Demographics	**N = 3320**	**N = 641**	**N = 2679**
	**unique participants**		
**May respondent, n (%)**	2584 (77.8)	503 (78.5)	2081 (77.7)
**Dec respondent, n (%)**	1581 (47.6)	327 (51.0)	1254 (46.8)
**Responded to both surveys, n (%)**	845 (25.5)	189 (29.5)	656 (24.5)
Age, years MEDIAN (IQR)	42.0 (34, 52)	45.0 (35, 56)	41.0 (34, 51)
**Age group, years, n (%)**			
18–29	392 (11.8)	55 (8.6)	337 (12.6)
30–49	1888 (56.9)	329 (51.3)	1559 (58.2)
50–64	925 (27.9)	204 (31.8)	721 (26.9)
65+	115 (3.5)	53 (8.3)	62 (2.3)
**Race/Ethnicity, n (%)**			
White	2719 (81.9)	521 (81.3)	2198 (82.0)
Black	147 (4.4)	21 (3.3)	126 (4.7)
Hispanic/Latino	201 (6.1)	34 (5.3)	167 (6.2)
Asian/Pacific Islander	151 (4.5)	40 (6.2)	111 (4.1)
Other/mixed/PNTA	102 (3.1)	25 (3.9)	77 (2.9)
**Residence Region, n (%)**			
Region 1 Northeast	772 (23.3)	151 (23.6)	621 (23.2)
Region 2 Midwest	779 (23.5)	146 (22.8)	633 (23.6)
Region 3 South	1369 (41.2)	290 (45.2)	1079 (40.3)
Region 4 West	400 (12.0)	54 (8.4)	346 (12.9)
**Professional Role, n (%)**			
Physician/PA/NP	695 (20.9)	229 (35.7)	466 (17.4)
Nurse (RN/LPN) [D]	945 (28.5)	74 (11.5)	871 (32.5)
Paramedic/EMT Other/Missing	1216 (36.6)	259 (40.4)	957 (35.7)
Administrative and research [D]	464 (14.0)	79 (12.3)	385 (14.4)
**Type of Healthcare Facility, n (%)**			
Hospital	1770 (53.3)	359 (56.0)	1411 (52.7)
Skilled Nursing Facility	138 (4.2)	18 (2.8)	120 (4.5)
Outpatient	451 (13.6)	66 (10.3)	385 (14.4)
Urgent care clinic/Emergency services	117 (3.5)	50 (7.8)	67 (2.5)
Other	844 (25.4)	148 (23.1)	696 (26.0)
**Healthcare worker location n (%)**			
High infection risk unit/ service	750 (22.6)	174 (27.1)	576 (21.5)
Other unit/service	1506 (45.4)	268 (41.8)	1238 (46.2)
Technical/peripheral clinical services	220 (6.6)	44 (6.9)	176 (6.6)
Non-clinical services	51 (1.5)	12 (1.9)	39 (1.5)
Other or Not reported	793 (23.9)	143 (22.3)	650 (24.3)
Work hours per week before the pandemic, n (%)*			
0 hr	61 (1.8)	8 (1.2)	53 (4.0)
1–19 hrs	210 (6.3)	47 (7.3)	163 (6.1)
20–39 hrs	1070 (32.3)	121 (18.9)	949 (35.5)
40–59 hrs	1792 (54.1)	394 (61.5)	1398 (52.3)
60+ hrs	180 (5.4)	71 (11.1)	109 (4.3)
Work hours in the past week, n (%)*			
0 hr	192 (5.8)	36 (5.6)	156 (5.8)
1–19 hrs	346 (10.4)	61 (9.5)	285 (10.6)
20–39 hrs	1021 (30.8)	125 (19.5)	896 (33.5)
40–59 hrs	1537 (46.3)	345 (53.8)	1192 (44.5)
60+ hrs	222 (6.7)	74 (11.5)	148 (5.5)
**Regularly care for a child or sick, elderly, or disabled person**	291 (8.7)	32 (5.0)	259 (9.7)

*N = 7 respondents missing from work hours prior to pandemic; N = 2 missing response to work hours in past week

### Primary outcome: Intention to leave

**Fig 2:**
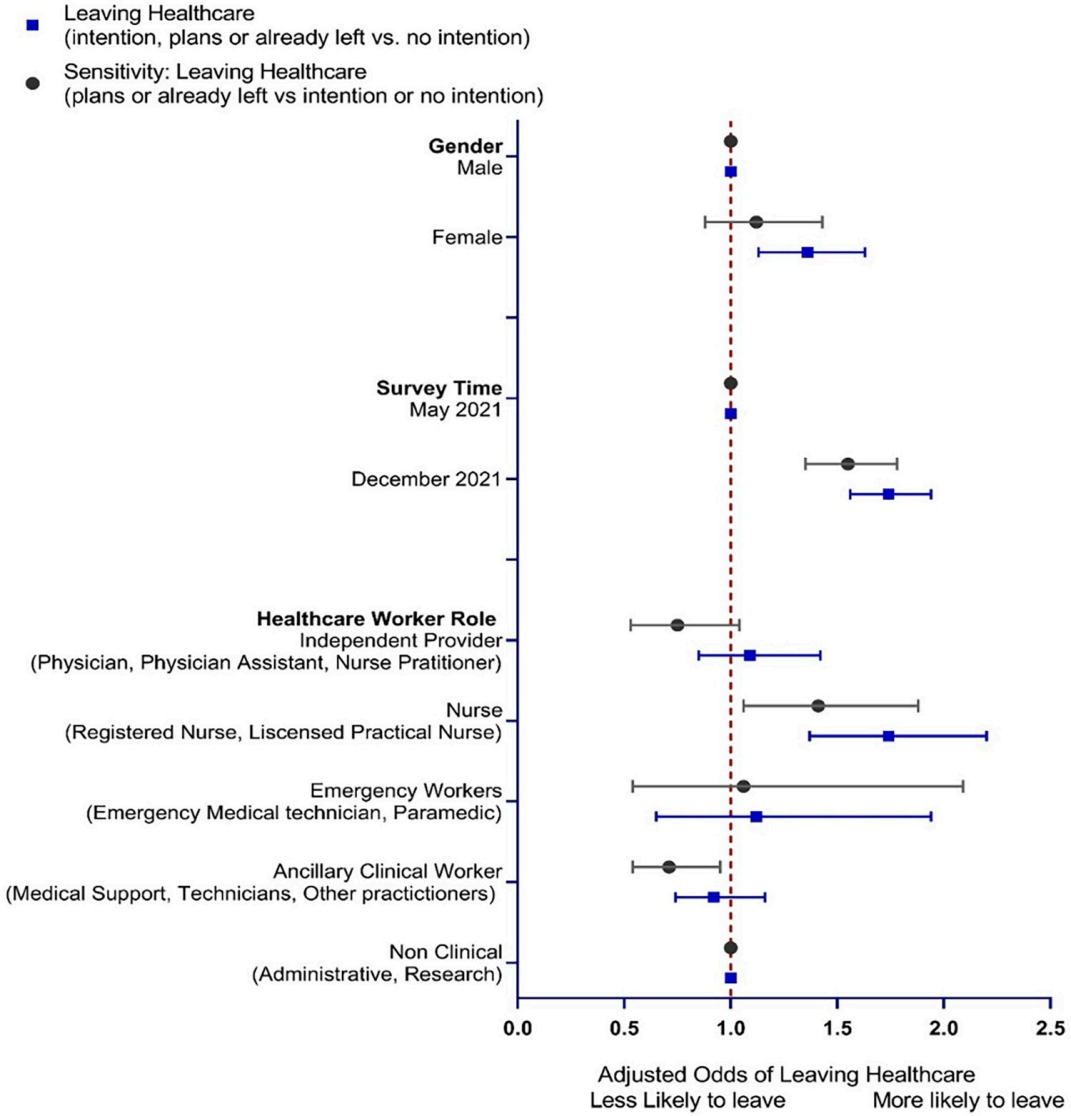
Odds of intention to leave healthcare by predictor variables.

Among the 4165 responses to either the May and December surveys, 1169/2584 (45.2%; May respondents) and 896/1581 (56.7%; December respondents) indicated they were thinking about, making plans, or had left their position or healthcare. Among the 4165 responses to either May or December surveys, female gender was associated with increased odds of intention to leave (42.2% males versus 51.4% females reported intent to leave; aOR 1.36 [1.13, 1.63]). Adjusted odds of intention to leave were higher among December responses compared with May responses (aOR 1.74, 95% CI 1.56, 1.94). Nurses had higher odds of intention to leave compared to administrative or research staff, who were the reference standard (aOR 1.74, 95% CI 1.37, 2.20) (**Table 2 and Fig 2**). Results of the mixed effects model that used an unstructured covariance structure were similar. (data not shown).

**Table 2:** Association of gender with healthcare worker intention to leave.

**Primary Analysis: left, actively planning or thinking about leaving versus no intention to leave**			
	**Intent to Leave n Yes/N Responses (%)**	**Unadjusted Odds Ratio (95% CI)**	Adjusted Odds Ratio* (95% CI)
**TIME OF SURVEY**			
MAY 2021	1,169/2,584 (45.2%)	Reference	Reference
DECEMBER 2021	896/1,581 (56.7%)	1.64 (1.48, 1.82)	1.74 (1.56, 1.94)
**GENDER**			
FEMALE	1,715/3,335 (51.4%)	1.49 (1.26, 1.76)	1.36 (1.13, 1.63)
MALE	350/830 (42.2%)	Reference	Reference
**WORKER ROLE**			
PHYSICIAN/PHYSICIAN ASSISTANT/ NURSE PRACTITIONER	431/905 (47.6%)	1.11 (0.88, 1.39)	1.09 (0.85, 1.42)
NURSES	713/1,159 (61.5%)	2.03 (1.64, 2.52)	1.74 (1.37, 2.20)
PARAMEDIC/EMERGENCY MEDICAL TECHNICIAN	59/113 (52.2%)	1.35 (0.88, 2.07)	1.12 (0.65, 1.94)
OTHER HEALTHCARE WORKER	610/1,415 (43.1%)	0.97 (0.79, 1.20)	0.92 (0.74, 1.16)
ADMIN/RESEARCH	252/573 (44.0%)	Reference	Reference
**Sensitivity analysis: left or actively planning versus thinking about leaving or no intention to leave**			
	**Intent to Leave n Yes/N Responses (%)**	**Unadjusted Odds Ratio (95% CI)**	**Adjusted Odds Ratio (95% CI)**
**TIME OF SURVEY**			
DECEMBER	348/1,581 (22.0%)	1.51 (1.32, 1.72)	1.55 (1.35, 1.78)
MAY	411/2,584 (15.9%)	Reference	Reference
**GENDER**			
FEMALE	621/3,335 (18.6%)	1.18 (0.94, 1.47)	1.12 (0.88, 1.43)
MALE	138/830 (16.6%)	Reference	Reference
**WORKER ROLE**			
PHYSICIAN/PHYSICIAN ASSISTANT/ NURSE PRACTITIONER	125/905 (13.8%)	0.68 (0.50, 0.93)	0.75 (0.53, 1.04)
NURSES	284/1,159 (24.5%)	1.41 (1.09, 1.83)	1.41 (1.06, 1.88)
PARAMEDIC/EMERGENCY MEDICAL TECHNICIAN	32/113 (28.3%)	1.54 (0.92, 2.58)	1.06 (0.54, 2.09)
OTHER HEALTHCARE WORKER	211/1,415 (14.9%)	0.77 (0.59, 1.01)	0.71 (0.54, 0.95)
ADMINISTRATION/RESEARCH	107/573 (18.7%)	Reference	Reference

^a^ Primary Analysis considers any intention to leave versus no intention to leave; sensitivity analysis considers intention to leave only those who have left and actively planning to leave versus thinking about leaving or no intention. Odds ratios and 95% confidence intervals (CI) reported.

^b^ Multivariable model that includes age, race/ethnicity, healthcare worker role, survey wave, family composition, self-reported caretaker status, healthcare facility, healthcare location, and weekly work hours.

### Sensitivity analysis: Modified intention to leave definition

We conducted a sensitivity analysis in which those persons who noted that they had thought about leaving but had not made any active plans to do so were included in the no intention to leave group. Results were consistent with the main results, but confidence intervals were wider (**Table 2** and **Fig 2**).

### Evaluation of burnout and moral injury

In the May survey, 76% of female compared with 69% of male respondents who expressed intention to leave had reported burnout as a contributor. In the December survey, 75% females compared with 71% for males who expressed intention to leave reported burnout. Among those who expressed intention to leave, one third of participants reported experience of moral injury, with similar levels reported by females and males (May: 35% in female, 34% in male; Dec: 33% in both female and male). Higher proportions of burnout and job-related moral injury were seen in the groups with intention to leave but no active plans compared to those who had already left or were actively planning to leave (**Table 3**).

**Table 3:** Job-related burnout and moral injury among those who report intention to leave healthcare.

	Intention but no plans	Planning/Already left
**May Survey**	n = 758	n = 411
Job-related Burnout, N (%)	603 (79.6)	268 (65.2)
Job-related moral injury, N (%)	270 (35.6)	134 (32.6)
**December Survey**	n = 548	n = 348
Job-related Burnout, N (%)	431 (78.6)	234 (67.2)
Job-related moral injury, N (%)	189 (34.5)	109 (31.3)

Note: Only respondents who noted an intention to Leave were asked about contributions of Burnout or Moral Injury

### Exploratory analysis

We conducted a univariate analysis which included caregiver status and healthcare worker role on intention to leave. In this model, we found that nurses were significantly associated with intention to leave as compared to physicians/PA/NP on both the primary and secondary intention to leave endpoints. Caregiver status was not significantly associated with intention to leave at either endpoint.

## Discussion

In this study of healthcare workers during the COVID-19 pandemic, we found that female gender was associated with a 36% higher odds of intention to leave compared with their male counterparts. We also report that compared to other provider types; nurses had 74% higher odds of intent to leave compared to most other health professionals. Respondents of both genders reported job-related burnout, with higher levels of burnout and moral injury reported in women compared to men. Those who had intention to leave but not made plans to do so reported the higher proportion of burnout and moral injury, compared to those who had already made plans or left, indicating low control of their work/job situation.

Our study is similar to other studies that explore the relationship between the COVID-19 pandemic and healthcare worker attrition, but advances what is known in that few have examined the impact of gender on intention to leave among a geographically diverse population and across multiple roles within the healthcare field [4, 17, 19–23]. Our study advances what is known about intention to leave healthcare among a geographically diverse population and across multiple roles within the healthcare field. Few if any other studies have utilized a national registry such as the HERO registry to examine these relationships and therefore are potentially less generalizable. Our study is similar to other studies that explore the relationship between the COVID-19 pandemic and healthcare worker attrition, but advances what is known in that few have examined the impact of gender on intention to leave among a geographically diverse population, in a longitudinal fashion and across multiple roles within the healthcare field [4, 17, 19–23].

Delaney et al. reported in a single U.S. health system that women and caregivers were more likely to consider leaving the healthcare workforce or reducing their working hours secondary to the COVID-19 pandemic [21]. We build on these findings, by reporting similar results across multiple healthcare systems and across varied healthcare worker roles. Waqas et al. conducted an online survey of 197 U.S. dermatologists in 2020 and reported that there was a greater impact of the COVID-19 pandemic on females compared to males in terms of ability to work, particularly with respect to childcare duties for those respondents with children. The Waqas study did not assess for intention to leave the field but rather an intention to modify work hours due to the pandemic. There are a few small international studies that examined intent to leave healthcare among diverse groups of healthcare workers but none were powered to adequately examine the impact of a specific job-role, such as nurses versus other provider types, on intention to leave [20, 26, 27]. Our study advances the literature by providing comparison among different healthcare worker roles and intention to leave.

Other studies have examined burnout among clinicians, and even prior to the pandemic, burnout rates were typically found to be higher in females as compared to males [28–31]. Other studies have examined the relationship of burnout, moral injury and intention to leave healthcare in the context of the COVID-19 pandemic [32, 33]. LeClaire et al. found that nurses were disproportionately impacted by burnout and moral injury as compared to physicians, and that those clinicians experiencing burnout had a two to four-fold greater odds of intention to leave their healthcare jobs [30]. Our analysis builds on these prior studies by allowing further examination of the layers of intention to leave or change healthcare roles beyond a binary analysis of intention to leave versus no intention to leave. We found that the highest proportions of burnout and moral injury were among those groups who had intention to leave but no active plans to do so. This is perhaps due to further distress which could result from feelings of lack of control over one’s work situation and/or inability to make a change in job despite burnout and/or moral distress.

This study is limited in part by the study’s sample selection, which was composed of individuals who registered to participate in the HERO registry. While all 50 states and the District of Columbia are represented, this sample should be considered a convenience sample and not necessarily nationally representative, as participants were recruited in large part from academic medical centers which are part of the National Patient-Centered Clinical Research Network (PCORnet). As noted in a prior HERO registry publication, our sample nonetheless had demographic features consistent with the CDC’s April 2020 report on more than 9000 healthcare workers infected with COVID-19 [24, 34]. Due to the nature of distribution of the survey, a response rate cannot be calculated, but studies of providers typically report a response rate of approximately 20% [33]. Since the HERO registry was voluntary, we assume that our response rate is at least 20%. Additionally, the baseline survey did not ask participants about their baseline intention to leave. Data collected before the COVID-19 pandemic reported that among physicians, intention to leave is approximately 20–30% [33]. Additionally, given that our data was collected in 2021 and noting that the overall percentage of respondents identifying themselves as caregivers was relatively low, it is possible that caregivers of young or elderly dependents had already left the healthcare workforce by the time that our data was collected.

Additionally, the questionnaire did not adequately capture the degree to which multiple stressors may have played a role in respondents’ consideration of intention to leave. Many factors may contribute to intention to leave or change their healthcare field for females including: promotion inequality, unequal pay, and other factors including caregiving during a pandemic. Important variables such as ages of the children in the household was not available and may be in the causal pathway between female gender and intention to leave healthcare. Prior studies have shown that females, particularly those married with children, bear the majority of home and childcare-related responsibilities [15]. We note that the majority of respondents were female, which is a limitation for conducting analyses which explore gender-differences. However, according to most recent U.S. Bureau of Labor Statistics, the majority of healthcare workers are female, and additionally, the majority of respondents in survey-based studies are also female [34, 35]. Thus, our sample is considered representative. Nonetheless, the predominantly female respondent base of our study is a limitation to what we can infer about male workers and their intention to leave healthcare during the COVID-19 pandemic. Finally, there were a small number of participants who answered both the May survey and December survey, which limits our ability to make inferences about longitudinal trends over the course of 2021.

In conclusion, we demonstrate that among this geographically and vocationally diverse group of healthcare workers, women had a higher odds of intending to leave or change their healthcare field than men and also had higher self-reported burnout and moral injury, which may be important contributors to their intent to leave. Further research is needed to understand the degree to which family-related stressors play a role in intention to leave, particularly in the context of a global pandemic. As a result of the COVID-19 pandemic, the healthcare system remains under stress. The downstream impact of the exodus of healthcare workers from the workforce is still being realized. In particular, there continue to be nursing shortages filled by an increase in the numbers of traveling nurses [36]. Traveling nurses, while filling these important gaps in patient care, demand higher salaries (leading to health system financial strain) and may have less input and investment into a health systems organizational culture. The downstream ramifications of these and other changes within the healthcare workforce remain areas in need of ongoing research.
